# Racial or Ethnic and Multimorbidity Differences in Functional Limitation Trajectories Among Older Americans

**DOI:** 10.1093/geroni/igaa057.1062

**Published:** 2020-12-16

**Authors:** Ana Quinones, Miriam Elman, Anda Botoseneanu, Jason Newsom, David Dorr, Corey Nagel, Heather Allore

**Affiliations:** 1 Oregon Health & Science University, Portland, Oregon, United States; 2 OHSU-PSU School of Public Health, Portland, Oregon, United States; 3 University of Michigan, Dearborn, Michigan, United States; 4 Portland State University, Portland, Oregon, United States; 5 University of Arkansas for Medical Sciences, Little Rock, Arkansas, United States; 6 Yale University, New Haven, Connecticut, United States

## Abstract

Racial/ethnic minority groups in the U.S. are at risk for greater co-existing chronic disease (multimorbidity) burden and experience greater functional limitations relative to non-Hispanic white peers. To target programs designed to preserve functional independence, this study aims to identify temporal trends of functional limitation among race/ethnic groups and within the context of multimorbidity. Data from the Health & Retirement Study (2000-2014, N=16,959, 65 years of age and older, community-dwelling adults) were used in generalized estimating equation (GEE) models to assess changes in functional limitations over time (combined activities of daily living [ADL] and instrumental activities of daily living [IADL], range 0-11). Models were adjusted for race/ethnicity (non-Hispanic black, Hispanic, non-Hispanic white), self-reported chronic disease categories (no/one, ≥2 somatic, somatic-depression; of arthritis, cancer, diabetes, heart disease, high depressive symptoms [CES-D8≥4], hypertension, lung disease, stroke), age at baseline, sex, body-mass index, education, partnered, net worth, and time. In adjusted GEE models, Hispanic and black respondents experience 1.4 times greater counts of functional limitations, respectively, relative to white respondents (incidence rate ratio [IRR]= 1.4, 95% CI[1.17, 1.66], IRR=1.4, CI[1.26, 1.61]); however, temporal trends were similar. With regard to multimorbidity categories, somatic or somatic-depression multimorbidity were each associated with 2.2 or 3.5 times greater functional limitations, respectively, relative to having no/one condition (IRR=2.2, CI[2.06, 2.39], IRR=3.5, CI[3.18, 3.74]). There are marked differences in functional limitation levels between minority ethnic and white groups, as well as among chronic disease combination groups, suggesting the need to intervene in middle-age to reduce disparities.

